# Safety of Ferric Citrate Hydrate in Pregnant Women With Iron Deficiency Anemia, Fetuses, and Newborns: A Real‐World, Observational, Post‐Marketing Surveillance Study

**DOI:** 10.1111/jog.70243

**Published:** 2026-03-25

**Authors:** Shinichiro Suzuki, Teruo Hashimoto, Yuri Okuda, Kyoko Ito, Sayaka Ito, Masahiko Shigeta, Yuki Goto, Eiji Horikawa, Noriaki Nishino, Ryoichi Yamada

**Affiliations:** ^1^ Pharmaceutical Division Japan Tobacco Inc. Chuo‐ku Tokyo Japan; ^2^ Medical Affairs Department Torii Pharmaceutical Co. Ltd. Chuo‐ku Tokyo Japan; ^3^ Pharmacovigilance Department Torii Pharmaceutical Co., Ltd. Chuo‐ku Tokyo Japan

**Keywords:** fetal physiologic changes in pregnancy, hematologic and clotting, nutrition, pharmacology, prenatal care

## Abstract

**Aim:**

This post‐marketing surveillance study determined the real‐world use and safety of ferric citrate hydrate (FC) in fetuses and pregnant women with iron deficiency anemia (IDA).

**Methods:**

Pregnant women with IDA were registered centrally and followed prospectively from the first FC administration until the end of pregnancy or at treatment discontinuation. Demographic characteristics, FC administration, and levels of iron‐ and anemia‐related parameters were collected. Newborns were followed for 7 days after birth.

**Results:**

Registration was from October 2021 to March 2023, and 149 women and 129 newborns were included for analysis. The mean (standard deviation; SD) age of the women was 32.1 (5.1) years, and 75.8% were at ≥ 28 weeks of gestational age at the first FC administration. The mean (SD) daily dose of FC was 494.2 (36.3) mg. FC treatment was continued until the end of pregnancy in 53 of 149 women (35.6%). The most frequent reason for the discontinuation of FC (96/149 women, 64.4%) was an improvement in IDA (68/149 women, 45.6%). Adverse drug reactions (ADRs) led to discontinuation in 4 of 149 women (2.7%). In 7 of 149 women (4.7%), 9 ADRs were found, including constipation (3/149, 2.0%), nausea (2/149, 1.3%), abdominal pain upper (1/149, 0.7%), diarrhea (1/149, 0.7%), hand dermatitis (1/149, 0.7%), and fetal death (1/149, 0.7%). No ADRs were found in newborns. Hemoglobin and serum ferritin levels were increased with FC treatment, but no signs of iron overload were reported.

**Conclusions:**

No previously unknown safety concerns were detected in women, fetuses, or newborns by FC treatment during pregnancy.

**Trial Registration:**

jRCT2031210322

## 1 Introduction

Anemia is commonly observed in pregnant women. The World Health Organization defines anemia in pregnant women as a hemoglobin level < 11.0 g/dL (first and third trimester) or 10.5 g/dL (second trimester) and estimates that approximately 37% of pregnant women worldwide are affected by anemia [1]. Anemia increases the risk of cesarean section, maternal and infant mortality, and adverse outcomes in newborns [1, 2].

During pregnancy, the circulating blood volume of mothers increases to supply nutrients and oxygen to the fetus by facilitating blood flow in the placenta, and the iron requirement increases to expand maternal erythrocytes, in addition to the iron requirement for the fetus and placenta [3, 4]. In many cases, dietary iron intake and iron stores are insufficient to meet this increased iron requirement during pregnancy. Iron deficiency accounts for most cases of anemia [2].

Iron replacement is one option to treat iron deficiency anemia (IDA) in pregnant women [4]. Oral iron preparations are recommended as first‐line drugs for the treatment of IDA and are effective in anemic pregnant women [5]. However, oral iron preparations often induce gastrointestinal adverse drug reactions (ADRs), such as nausea, abdominal pain, diarrhea, and constipation, which might lead to non‐adherence [6].

Ferric citrate hydrate (FC) is an iron‐based phosphate binder that was approved in 2014 in Japan to treat hyperphosphatemia in patients with chronic kidney disease. In addition, FC demonstrated efficacy and safety in patients with IDA with or without chronic kidney disease, and it was approved for the treatment of IDA in 2021 in Japan [7, 8]. FC administration was associated with a significantly lower incidence of nausea and vomiting during treatment than conventional oral iron preparations [7]. In real‐world situations, FC is also prescribed to pregnant women with IDA. However, its safety in pregnant women, fetuses, and newborns has not been investigated in clinical studies and requires further study.

In the current study, we performed a post‐marketing surveillance analysis focusing on the safety of FC in women and fetuses when administered to pregnant women with IDA.

## 2 Methods

### 2.1 Study Design

This was a prospective observational study to evaluate the safety of FC in pregnant women, fetuses, and newborns. The study was conducted in compliance with the ministerial ordinance on good post‐marketing study practice of the Ministry of Health, Labour and Welfare of Japan, and in accordance with the Declaration of Helsinki. Women were asked to provide informed consent to participate in the study. The planned registration period of patients was from October 1, 2021 to March 31, 2023, and the survey forms were collected from October 1, 2021 to September 30, 2024. This study was registered at the Japan Registry of Clinical Trials (jRCT2031210322).

### 2.2 FC Treatment

FC (Riona, Japan Tobacco Inc., Tokyo, Japan; distributed by Torii Pharmaceutical Co., Ltd. Tokyo, Japan) is a 250 mg tablet containing approximately 60 mg of elemental ferric iron (Fe^3+^). FC has been approved in Japan to treat IDA and is administered within general clinical practice as the Japanese package insert recommends: orally at a dose of two tablets (500 mg as FC, approximately 120 mg as Fe^3+^) once a day immediately after a meal. The dose was adjusted according to the level of anemia and clinical status of patients, with a maximum dose of two tablets at once, twice a day (1000 mg as FC, approximately 240 mg as Fe^3+^).

In Japan, 36 gynecological and/or obstetrics institutions were contracted to participate in the study. Eligible participants were pregnant women who were treated with FC because of IDA diagnosed based on the physician's experience; who were registered by treating physicians within 14 days after the first treatment with FC; who provided informed consent; and who underwent registration during the contracted period. No inclusion criteria were set regarding the baseline levels of hemoglobin or serum ferritin. For newborns, consent was provided by their mothers. Patients who were previously treated with FC were excluded. Patients were registered centrally and followed prospectively using clinical survey forms. The observation period for women was defined as the period from the first FC administration until the end of pregnancy. In cases where FC was terminated before the end of pregnancy, the observation period was until the last FC treatment. The follow‐up period for newborns was from birth to 7 days after birth. Information about the women, including demographic characteristics, dosing, and treatment period of FC, and means of delivery, was collected during the observation period. Hemoglobin, serum ferritin, and red blood cell counts were collected as iron‐ and anemia‐related laboratory test values, when available. Information about the newborns, including demographic characteristics and Apgar scores, was collected during the follow‐up period.

### 2.3 Safety

Safety information was collected from women and newborns. All women whose survey forms were collected and contained information from the first FC administration until the end of pregnancy or discontinuation of FC, and all newborns whose follow‐up survey forms were collected with information from birth until 7 days after birth, were included in the safety analysis set.

All ADRs from women and newborns were collected. ADRs in women were collected during the observation period and up to 14 days after the last day of FC administration when FC treatment was discontinued before the end of pregnancy. ADRs in newborns were collected during the follow‐up period. In this study, no ADRs of special interest were defined. Laboratory test values for iron‐ and anemia‐related parameters were collected from women to monitor IDA (i.e., hemoglobin, serum ferritin, and red blood cell counts). To record ADRs, the terms in MedDRA, version 26.1, were used.

### 2.4 Statistics

In this study, we planned to include 100 participants. In previous Phase II–III clinical trials in patients with IDA [9], pregnant women were excluded. In the IDA population in these clinical studies, the most common ADRs were diarrhea (76/465 patients, 16.3%), nausea (45/465 patients, 9.7%), and constipation (16/465 patients, 3.4%). With the frequency of an ADR assumed as 3% in pregnant women, the ADR could be detected in at least 1 of 100 participants with a 95% possibility. From the period of October 1, 2021 to March 31, 2023, registration of 100 pregnant women was considered feasible, and 100 participants would allow us to evaluate whether the frequency of ADRs in pregnant women was equivalent to or higher than that in patients included in the previous clinical studies. Therefore, the target number of participating women was set as 100.

Summary statistics were used to describe the demographic characteristics of the women and newborns. Time‐course changes in laboratory test values were depicted in graphs for the observation period.

## 3 Results

### 3.1 Demographic Characteristics of Women in This Study

**FIGURE 1:**
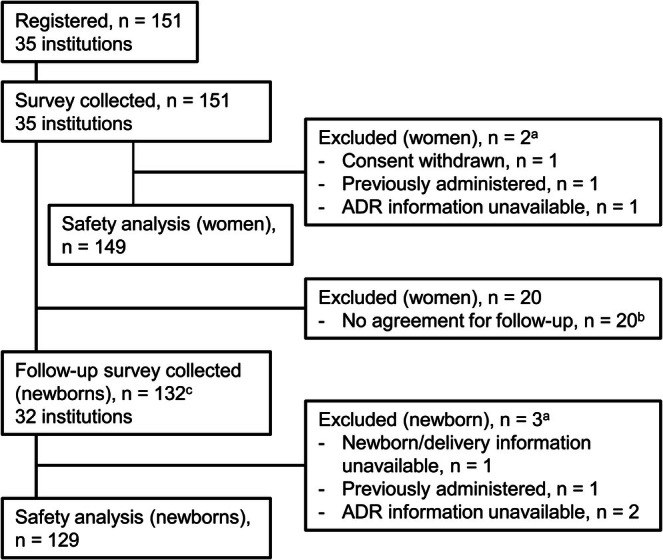
Patient flow diagram. ^a^One patient with multiple reasons was possible. ^b^Including one case, in which consent was withdrawn by the study participant (*n* = 1). ^c^Including multiple births (*n* = 2). ADR, adverse drug reaction.

Overall, 151 women from 35 institutions were registered, and 149 women and 129 newborns were included in the safety analysis set. A flowchart of study participants is shown in Figure 1. The planned number of participants was achieved early, and the survey was completed on January 23, 2024.

The demographic characteristics of the women at the first administration of FC are summarized in Table 1. The mean age ± standard deviation (SD) was 32.1 ± 5.1 years. The highest proportion of women was≥ 28 weeks of gestational age at the first FC administration (113/149 women, 75.8%). At the first FC administration, 130 of 149 women (87.2%) had a hemoglobin value < 11.0 g/dL (cutoff to define anemia), and 13 of 149 women (8.7%) had a hemoglobin value ≥ 11.0/dL. Serum ferritin value was < 12.0 ng/mL (cutoff to define IDA) in 29 of 149 women (19.5%) and ≥ 12.0 ng/mL in 11 of 149 women (7.4%), and the value was missing in 109 of 149 (73.2%). Comorbidities were present in 36 of 149 women (24.2%). The most common comorbidity was constipation (10/149 women, 6.7%), followed by premature labor (9/149, 6.0%), hypoparathyroidism, uterine leiomyoma, gestational diabetes (3/149, 2.0% each), allergic rhinitis, and chronic gastritis (2/149, 1.3% each).

**TABLE 1:** Demographic characteristics of women at the first administration of ferric citrate hydrate.

	*n* (%)^a^
Total number of women in the safety analysis set	149 (100.0)
Age, years	
Mean (SD)	32.1 (5.1)
Median (min, max)	33.0 (19, 42)
< 20	1 (0.7)
≥ 20, < 30	45 (30.2)
≥ 30, < 40	97 (65.1)
≥ 40, < 50	6 (4.0)
Gestational age, weeks	
< 14	5 (3.4)
≥ 14, < 28	31 (20.8)
≥ 28	113 (75.8)
Comorbidities	
Absent	113 (75.8)
Present	36 (24.2)
Hemoglobin, g/dL	
< 11.0	130 (87.2)
≥ 11.0	13 (8.7)
Unknown	6 (4.0)
Serum ferritin, ng/mL	
< 12.0	29 (19.5)
≥ 12.0	11 (7.4)
Unknown	109 (73.2)

Abbreviations: max, maximum; min, minimum; *n*, number; SD, standard deviation.

^a^ Otherwise as noted.

Anemia was previously treated in 17 of 149 women (11.4%) with sodium ferrous citrate (11/149, 7.4%), dried ferrous sulfate (3/149, 2.0%), saccharated ferric oxide (2/149, 1.3%), or ferrous fumarate (1/149, 0.7%). Concomitant medication was used in 45 of 149 women (30.2%). The main concomitant medications were magnesium oxide (11/149, 7.4%), ritodrine hydrochloride (9/149, 6.0%), sodium picosulfate hydrate, and fursultiamine/B2/B6/B12 (4/149, 2.7% each).

### 3.2 FC Treatment

The daily dose of FC was ≥ 500 and < 1000 mg in 145/149 women (97.3%), and < 500 mg in 4 of 149 women (2.7%). No woman received ≥ 1000 mg. The administration dose was similar in women of different gestational ages (Table 2). Overall, 53 of 149 women (35.6%) continued FC until the end of pregnancy, and 96/149 women (64.4%) discontinued FC, including 47 women within 4 weeks and 34 women 4–8 weeks after the initiation of FC treatment (Table 3). The mean ± SD administration period was 38.1 ± 32.9 days in all women, 45.8 ± 24.9 days, 69.5 ± 50.9 days, and 29.2 ± 19.0 days in women who started FC with a gestational age of < 14, ≥ 14 and < 28, and ≥ 28 weeks, respectively (Table 2). The total period of FC treatment was < 4 weeks in 64 women (43.0%), ≥ 4 and < 8 weeks in 55 women (36.9%), and ≥ 8 and < 12 weeks in 17 women (11.4%). The most frequent reason for discontinuation was an improvement in IDA (68/149 patients, 45.6%). Overall, 4 of 149 women (2.7%) discontinued FC because of ADRs. The gestational age of all women who discontinued FC because of ADRs was ≥ 28 weeks at the first FC administration.

**TABLE 2:** Administration of ferric citrate hydrate by gestational age at the first administration of ferric citrate hydrate.

	Total	Gestational age, weeks		
		< 14	≥ 14, < 28	≥ 28
Number of women in the safety analysis set	149	5	31	113
Daily dose, mg				
Mean (SD)	494.2 (36.3)	500.0 (0.0)	496.4 (20.0)	493.4 (40.4)
Median (min, max)	500 (250, 500)	500 (500, 500)	500 (389, 500)	500 (250, 500)
Administration period, days				
Mean (SD)	38.1 (32.9)	45.8 (24.9)	69.5 (50.9)	29.2 (19.0)
Median (min, max)	28.0 (1, 181)	41.0 (25, 88)	65.0 (7, 181)	28.0 (1, 82)

Abbreviations: max, maximum; min, minimum; SD, standard deviation.

**TABLE 3:** Administration period and discontinuation of ferric citrate hydrate.

	Administration period, weeks							
	< 4	≥ 4, < 8	≥ 8, < 12	≥ 12, < 16	≥ 16, < 20	≥ 20, < 24	≥ 24, < 28	Total, *n* (%)
Number of women in the safety analysis set	149	85	30	13	9	3	2	149 (100.0)
Administration continued until the end of pregnancy, *n*	17	21	9	1	2	1	2	53 (35.6)
Discontinuation, *n*	47	34	8	3	4	0	0	96 (64.4)
Adverse drug reactions, *n*	3	1	0	0	0	0	0	4 (2.7)
Effectiveness not satisfactory, *n*	4	1	0	0	0	0	0	5 (3.4)
Improvement of iron deficiency anemia^a^, *n*	29	27	7	2	3	0	0	68 (45.6)
Hospital transfer or no visit, *n*	4	2	0	0	0	0	0	6 (4.0)
Others, *n*	8	3	1	1	1	0	0	14 (9.4)

Abbreviation: *n*, number.

^a^ The criteria used for determining improvement and discontinuation were selected by the physician and based on the physician's experience.

### 3.3 Demographic Characteristics of Newborns

The characteristics of newborns are summarized in Tables 4 and 5. There were 60 of 129 males (46.5%) and 69 of 129 females (53.5%). A large proportion of newborns were delivered naturally (98/129 newborns, 76.0%), followed by cesarean section (18/129, 14.0%), vacuum extraction (9/129, 7.0%), and forceps delivery (2/129, 1.6%). Almost all newborns were normal (126/129, 97.7%) except for one case each of premature birth/low birth weight, low birth weight, and neonatal asphyxia (1/129, 0.8%, each). No congenital anomaly or stillbirth was reported. An Apgar score of ≥ 7 was recorded 1 min after birth in 127 of 129 newborns (98.4%) and 5 min after birth in 129 of 129 newborns (100%). The heights and weights (mean ± SD) of the newborns were 49.3 ± 1.9 cm and 3.12 ± 0.37 kg, respectively.

**TABLE 4:** Demographic characteristics of newborns.

	*n* (%)
Total number of newborns in the safety analysis set	129 (100.0)
Sex	
Male	60 (46.5)
Female	69 (53.5)
Means of delivery	
Natural childbirth	98 (76.0)
Cesarean section	18 (14.0)
Vacuum extraction	9 (7.0)
Forceps delivery	2 (1.6)
Others	2 (1.6)
Birth status	
Normal newborns	126 (97.7)
Others	3 (2.3)
Apgar score 1 min after birth	
≥ 7	127 (98.4)
4–6	1 (0.8)
≤ 3	1 (0.8)
Apgar score 5 min after birth	
≥ 7	129 (100.0)
4–6	0 (0.0)
≤ 3	0 (0.0)

Abbreviation: *n*, number.

**TABLE 5:** Demographic characteristics of newborns by gestational ages at the first administration of ferric citrate hydrate.

	Total	Gestational age, weeks		
		< 14	≥ 14, < 28	≥ 28
Height, cm				
Number of newborns in the safety analysis set	127^a^	3	23	101
Mean (SD)	49.3 (1.9)	48.7 (1.3)	49.6 (2.2)	49.3 (1.8)
Median (min, max)	49.9 (43, 55)	48.6 (48, 50)	50.5 (45, 53)	49.8 (43, 55)
Weight, kg				
Number of newborns in the safety analysis set	129	3	23	103
Mean (SD)	3.12 (0.37)	2.92 (0.24)	3.12 (0.41)	3.12 (0.37)
Median (min, max)	3.14 (1.5, 4.1)	3.00 (2.7, 3.1)	3.11 (2.3, 4.1)	3.15 (1.5, 4.1)

Abbreviations: max, maximum; min, minimum; SD, standard deviation.

^a^ Among 129 newborns, data from two newborns were not available because of their transfer to other hospitals.

### 3.4 ADRs

Nine ADRs were noted in 7 of 149 women (4.7%). The most frequently reported ADRs were gastrointestinal disorders (6/149 women, 4.0%), including constipation (3/149 women, 2.0%), nausea (2/149 women, 1.3%), abdominal pain upper (1/149 women, 0.7%), and diarrhea (1/149 women, 0.7%) (Table 6). All women who experienced ADRs recovered. No ADRs related to iron overload were reported. No ADRs were found in newborns.

**TABLE 6:** Adverse drug reactions.

	*n* (%)
Number of women in the safety analysis set	149 (100)
Adverse drug reactions	7 (4.7)
Gastrointestinal disorders	6 (4.0)
Constipation	3 (2.0)
Nausea	2 (1.3)
Abdominal pain upper	1 (0.7)
Diarrhea	1 (0.7)
Skin and subcutaneous tissue disorders	1 (0.7)
Hand dermatitis	1 (0.7)
Pregnancy, postpartum, and perinatal conditions	1 (0.7)
Fetal death^a^	1 (0.7)
Number of newborns in the safety analysis set	129 (100)
Adverse drug reactions	0 (0.0)

Abbreviation: *n*, number.

^a^ Fetal death was counted as an adverse drug reaction in women, not newborns, by the reporting physician because it occurred in the uterus, and the fetus was not subjected to follow‐up.

One of the nine ADRs in women was fetal death (1/149 women, 0.7%). Fetal death was counted as an ADR in women, not newborns, by the reporting physician, and the fetus was not subjected to follow‐up. The woman was in her early 30s, and she had no notable medical history or comorbidities. FC administration at a dose of 500 mg was started at 12 weeks of gestational age because of IDA. Fetal death was confirmed at 4 weeks and 4 days after the first FC administration (at 16 weeks and 4 days of gestational age). FC treatment was continued for 5 weeks and 5 days after the first FC administration, and an improvement in IDA was confirmed 11 weeks after the first FC administration. No investigations to determine the cause of fetal death, such as an autopsy, were carried out, and the physician reported that the causal relationship between fetal death and FC was unknown.

### 3.5 Laboratory Test Values for Iron‐ and Anemia‐Related Parameters

**FIGURE 2:**
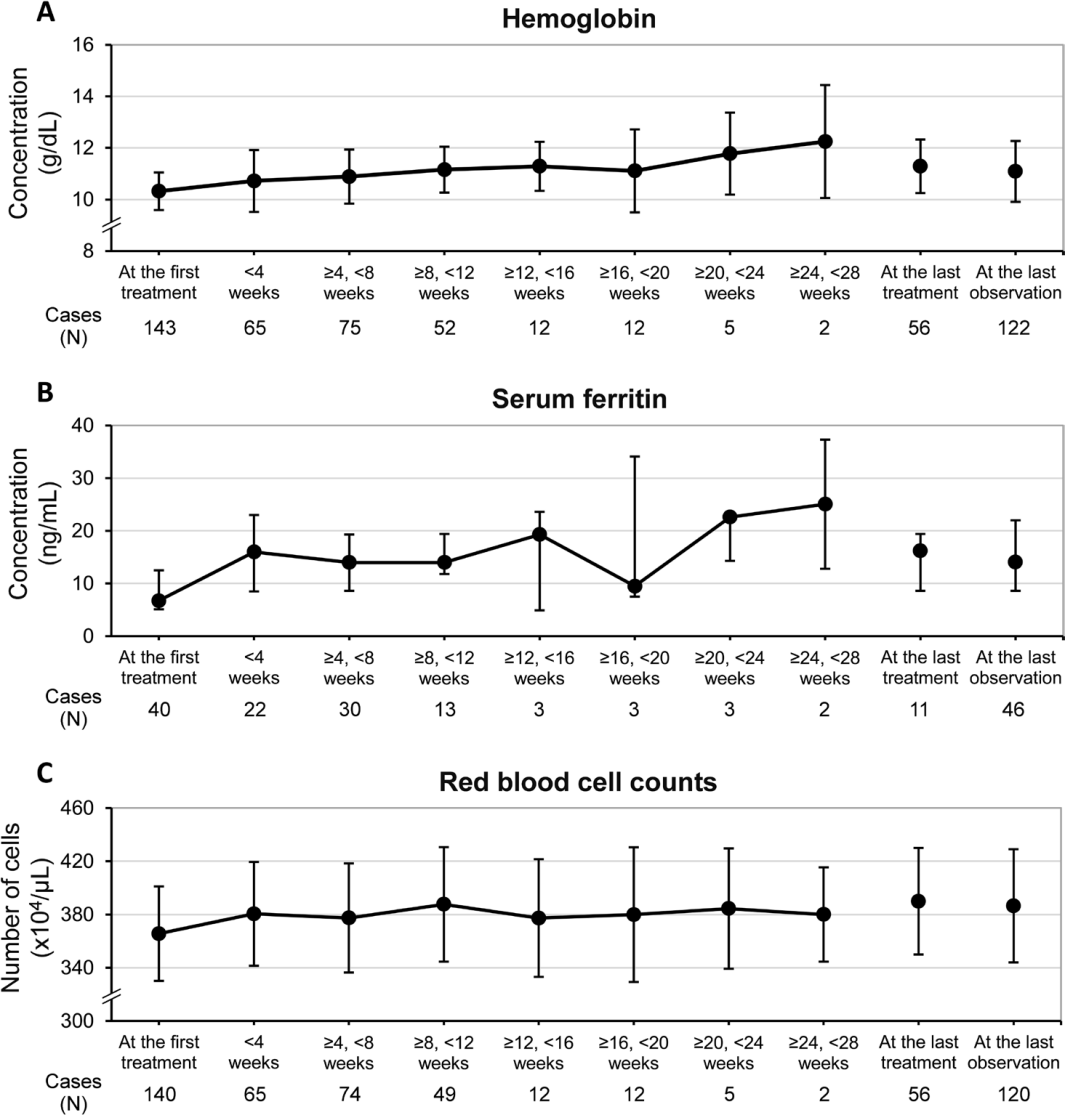
Time‐course changes in laboratory test values. (A) Hemoglobin, mean ± standard deviation. (B) Serum ferritin, median ± the first and third quartiles. (C) Red blood cell counts, mean ± standard deviation.

Laboratory test values for iron‐ and anemia‐related parameters (hemoglobin, serum ferritin, and red blood cell counts) are plotted in Figure 2. Hemoglobin values (mean ± SD) were 10.32 ± 0.73 g/dL at the first FC administration (*n* = 143) and 11.09 ± 1.18 g/dL at the last observation (*n* = 122). The mean changes ± SD from baseline were 0.78 ± 1.14 g/dL (*n* = 118), and the highest value throughout the observation period was 14.00 g/dL (Figure 2A). The median (first quartile, third quartile) values of serum ferritin were 6.75 ng/mL (5.10, 12.50) at the first FC administration (*n* = 40), and 14.05 ng/mL (8.60, 22.00) at the last observation (*n* = 46). The median change from baseline (first quartile, third quartile) was 2.60 ng/mL (0.00, 15.00) (*n* = 30). The highest value throughout the observation period was 345.6 ng/mL (Figure 2B), and no woman had a value that exceeded 500.0 ng/mL, the cut‐off value for iron overload in Japan [10]. Red blood cell counts (mean ± SD) were 365.6 ± 35.5 × 10^4^/μL at the first FC administration (*n* = 140) and 386.5 ± 42.5 × 10^4^/μL at the last observation (*n* = 120). The mean change ± SD from baseline was 22.1 ± 39.5 × 10^4^/μL (*n* = 116), and the highest value throughout the observation period was 505 × 10^4^/μL (Figure 2C). No notable abnormal increase or decrease was reported in any of these laboratory test values.

**FIGURE 3:**
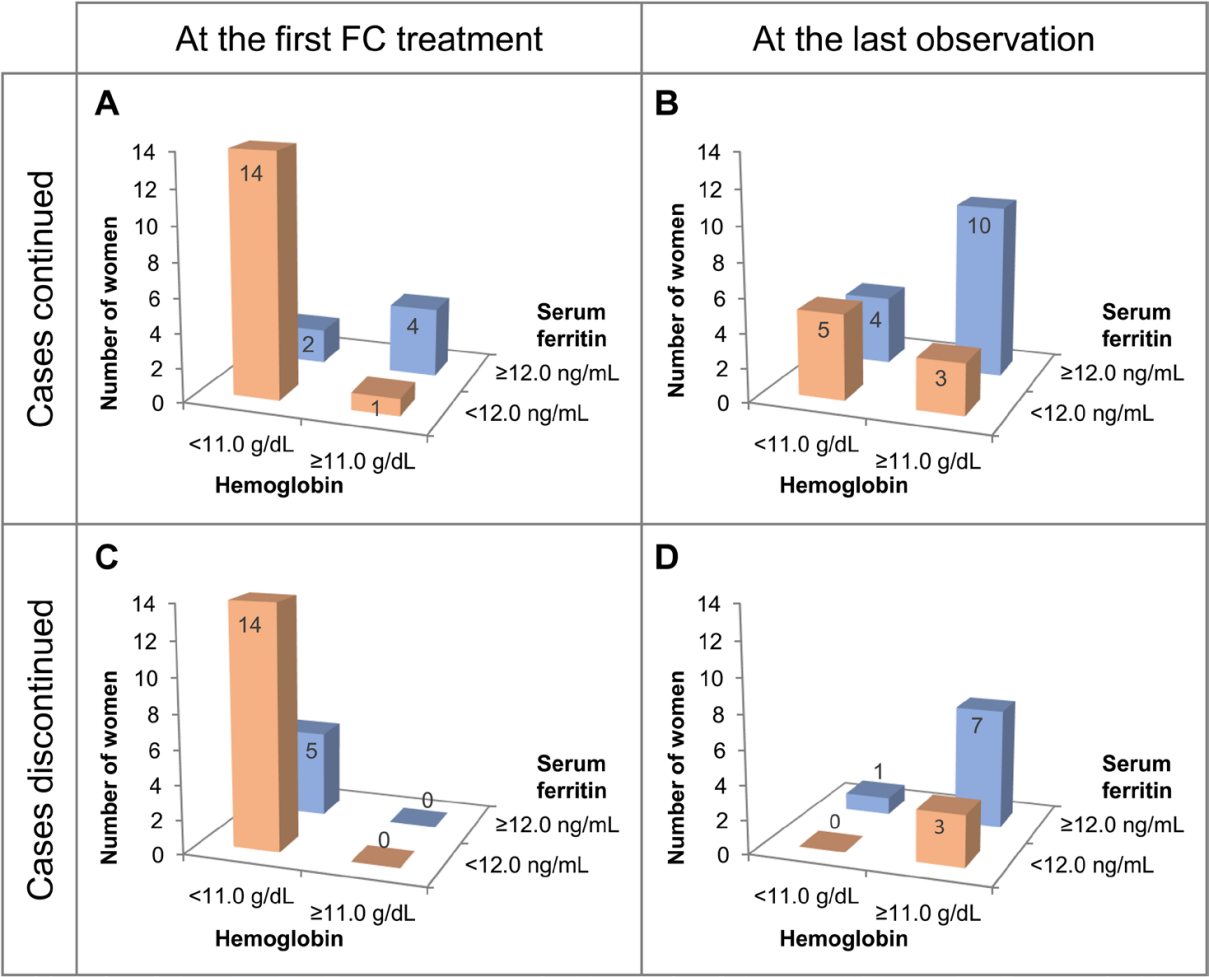
Changes in hemoglobin and serum ferritin levels. Distribution of women, stratified by categories including under and over anemia cut‐off values of hemoglobin (11.0 g/dL) and serum ferritin (12.0 ng/mL). (A, B) Women who continued FC until the end of pregnancy. (C, D) Women who discontinued FC before the end of pregnancy. (A, C) At the first FC administration. (B, D) At the last observation timepoint. FC, ferric citrate hydrate.

Figure 3 shows the distributions of women stratified in categories under and over the anemia cut‐off values of hemoglobin (11.0 g/dL) and serum ferritin (12.0 ng/mL) at the first FC administration and at the last observation timepoint (Figure 3). In both groups of women who continued FC until the end of pregnancy (Figure 3A,B) and who discontinued FC before the end of pregnancy (Figure 3C,D), the highest proportion of women at the first FC administration was under the anemia cut‐off values of hemoglobin and serum ferritin (Figure 3A,C). Hemoglobin and serum ferritin values increased during the treatment in the continued and discontinued groups, and at the last observation timepoint, the highest proportion of women was over the anemia cut‐off values of both parameters (Figure 3C,D).

## 4 Discussion

In this study, the safety of FC administered to pregnant women with IDA was evaluated in women, fetuses, and newborns. No new notable safety concerns were found.

ADRs were reported in seven women (4.7%), and six women developed gastrointestinal disorders. Generally, oral iron preparations are known to cause gastrointestinal ADRs, and FC has been considered to cause fewer gastrointestinal ADRs than ferrous iron preparations [7]. The types of gastrointestinal ADRs observed in this study were in accordance with the previously known ADRs with FC treatment. For example, among Japanese preapproval clinical trials including 465 patients with IDA, the most frequent ADRs were gastrointestinal disorders that developed in 125 cases (25.8%), with the main occurrences being diarrhea in 76 cases (16.3%), nausea in 45 cases (9.7%), and constipation in 16 cases (3.4%) [9]. Similarly, diarrhea (0.7%), constipation (2.0%), and nausea (1.3%) were noted in pregnant women in our study. The gastrointestinal ADRs were, however, less frequently observed in this study (4.7%) than in clinical studies (25.8%). The difference may be attributed to the study design: the present study was a post‐marketing surveillance study, in which AEs tend to be underreported [11]. In addition, because the inclusion criteria used in the present study were not as strict as those used in the clinical studies, it is not possible to compare ADR frequencies directly.

Hemoglobin and serum ferritin values were increased by FC treatment in the current study, but there were no signs of iron overload. Mean hemoglobin value was increased from 10.32 g/dL at baseline to 11.09 at the last observation with FC treatment (mean daily dose, 494.2 mg, approximately 118.6 mg of Fe^3+^). This increase was similar to that in a post‐marketing surveillance study for sodium ferrous citrate in pregnant women, where administration of 100 mg of sodium ferrous citrate, containing 100 mg of elemental ferrous iron (Fe^2+^), increased from 10.1 g/dL (at baseline) to 11.0 g/dL (at 4 weeks) [12].

There was one fetal death (0.7%), which was noted as a serious ADR. No investigations to determine the cause were carried out, and the physician reported that the causal relationship between fetal death and FC was unknown. As a reference, the Japanese national statistics reported that spontaneous fetal death rates were 0.96% in 2023 and 0.94% in 2022 [13]. In this study, 97.7% of 126 neonates were normal newborns, and no ADRs were reported for them. Studies using other iron preparations showed a similar safety profile. For example, the package insert of an iron preparation (sodium ferrous citrate) mentions follow‐up surveys of fetal and neonatal outcomes in post‐marketing studies that did not identify any particular safety issues for pregnant women or newborns [14]. A pilot study comparing the dosing of ferrous sulfate once per day, once every other day, and three times per week reported miscarriage, defined as a spontaneous loss of pregnancy before 24 weeks of gestational age, in 2 of 273 women (0.7%) in total, including in 1 of 86 women (1.2%) administered ferrous sulfate once per day, in 1 of 95 women (1.1%) administered ferrous sulfate once every other day, and 0 of 92 women (0.0%) administered ferrous sulfate three times per week [15]. Accordingly, the risk of FC treatment for pregnant women, fetal development, and delivery could be considered low, taking the Japanese national statistics into account, and comparable with other oral iron preparations, such as ferrous sulfate.

This study had several limitations. It was a single‐arm observational study and had no active or placebo controls for comparison; the number of included participants was small; and some information, including serum ferritin values, was incomplete or missing, reflecting the current real‐world clinical practice of treating pregnant women with IDA in Japan, in which physicians may use their discretion to determine the necessity of measuring ferritin values. Because of the nature of post‐marketing surveillance studies using real‐world data, there were no strict inclusion criteria, and the study population included women with hemoglobin values ≥ 11.0 g/dL at the first FC administration. It is possible that not all ADRs were detected because only ADRs reported on the surveillance forms were collected. Many women were at ≥ 28 weeks of gestational age (113/149 women, 75.8%), and we only included five participants who started FC at < 14 weeks of gestational age, which resulted in a short administration period of FC.

In conclusion, our post‐marketing surveillance study of pregnant women with IDA treated with FC detected no notable safety issues in women, fetuses, or newborns. The results indicate that although the known risk of iron overload and gastrointestinal ADRs exists, these risks can be well controlled by careful monitoring.

## Author Contributions

**Shinichiro Suzuki:** conceptualization, methodology, investigation, formal analysis, writing – original draft, visualization, writing – review and editing. **Teruo Hashimoto:** conceptualization, methodology, investigation, formal analysis, writing – original draft, visualization, writing – review and editing. **Yuri Okuda:** formal analysis, writing – original draft, visualization writing – review and editing. **Kyoko Ito:** formal analysis, writing – original draft, visualization writing – review and editing. **Sayaka Ito:** formal analysis, writing – original draft, visualization writing – review and editing. **Masahiko Shigeta:** conceptualization, methodology, investigation, formal analysis, writing – original draft, visualization, writing – review and editing. **Yuki Goto:** project administration, supervision, writing – review and editing. **Eiji Horikawa:** project administration, supervision, writing – review and editing. **Noriaki Nishino:** project administration, supervision, writing – review and editing. **Ryoichi Yamada:** project administration, supervision, conceptualization, methodology, investigation, formal analysis, writing – original draft, visualization, writing – review and editing.

## Funding

This work was supported by Japan Tobacco Inc., and Torii Pharmaceutical Co., Ltd.

## Disclosure

An earlier version of this article was presented at the 39th Japan Society for Menopause and Women's Health, held in Utsunomiya, Japan, on November 10–11, 2024.

## Ethics Statement

The study was conducted in compliance with the ministerial ordinance on good post‐marketing study practice of the Ministry of Health, Labour and Welfare of Japan and in accordance with the Declaration of Helsinki.

## Consent

Women were asked to provide informed consent to participate in the study.

## Conflicts of Interest

Shinichiro Suzuki, Teruo Hashimoto, Yuri Okuda, Yuki Goto, and Ryoichi Yamada were employees of Shionogi & Co. Ltd. (formerly Japan Tobacco Inc.). Kyoko Ito, Sayaka Ito, Masahiko Shigeta, Eiji Horikawa, and Noriaki Nishino were employees of Torii Pharmaceutical Co., Ltd. This surveillance study was funded by Japan Tobacco Inc., and Torii Pharmaceutical Co., Ltd.

## Data Availability

Sharing of the anonymized data of individual participants from this study with third parties, excluding health authorities, is prohibited in the contract between study sites and Japan Tobacco Inc./Torii Pharmaceutical Co., Ltd.
